# Supplementary material for: Color-complexity enabled exhaustive color-dots identification and spatial patterns testing in images
Source: PLoS One. 2021 May 11;16(5):e0251258. doi: 10.1371/journal.pone.0251258 (PMC8112684; doi:10.1371/journal.pone.0251258)
Supplement: Attachment — Submitted filename: VT-2021-PONE-D-20-32597.pdf [file pone.0251258.s001.pdf]

Review for "*Color-complexity enabled exhaustive color-dots identification and spatial patterns testing in images*" of Author Hsieh Fushing in University of California at Davis, CA USA.

A) Summary for the work

- In the paper, the authors want to perform "*color-dots identification*" and "*spatial patterns testing*" in digital images based on their concept "*Color complexity*".
- The authors also want to apply this study in the case of precision agriculture, where UAVs are used for spraying. Then homogeneity and distribution of sprayed liquid should be identified exactly to enhance the efficiency of UAVs activities.
- The homogeneity and distribution of sprayed liquids are described by the spatial locations of color-dots in a test paper imaged into a digital photo. It means that the first homogenous distribution is understood and researched as 2D spatial location distribution as its appearance. The second homogeneity is understood and researched as the homogeneity, amount, and concentration of chemical compound and is reflected by its color.
- The physical color is understood by the authors as the matrix of RGB or HSV.
- The authors have also given the concept "*color-complexity*" and based on a "*low color complexity*" for their work.
- They have built MST structure and its distance distribution. Herewith, they have created the algorithms for 2D-spatial uniformness and color identification.
- 5 images are taken from GEOSTAR Technology Inc for their work.
- They have concluded that:
    - *Both color identifications and testing 2D spatial uniformness via MST of 5 images are satisfactory.*
    - *Color identification based on the data-driven computation is effective and practical than other mentioned methods (OpenCV, Contour).*
    - *The key of the effectiveness is low color-complexity (their concept).*
    - *MST structure and distance distribution are new and essential summarizing pattern information of spatial data.*
    - *They propose to combine the results from both RGB and HSD under distinct scales to solve the shading in images and shading effects.*

B) Advantages of this work

- The authors are quite strong on the aspect of machine learning (ML) and data science (DS).
- The application idees of ML and DS are quite interesting and practical in precision agriculture using UAV based on color-dots and 2D spatial location.
- The algorithms given and applied with MST structure and distance distribution are very good. The idee of MST structure and its distance distribution are very practical.
- 5 images were analyzed quite well.
- The idees of (1) using color parameter or adjusted color parameters as the indicators of chemical amount and homogeneity (understood as color identification with low "*color complexity*") and (2) 2D spatial location are very interesting and very good.

C) Disadvantages and relevant questions

Although there are the strong points like the above mentions, the authors are quite weak on the aspect color science. They have also admitted that they are not strong in this vision and do not want to go deeply in the direction. But that is the right problem of this paper. When they do not understand

correctly or enough deeply on the color science, their built algorithms and conclusions are too subjective and quite hurry. Consequently, there are quite many relevant questions for it.

1) Comment 1 - The concept "*Color-Complexity*": Where does this concept come from? It has just introduced at the first time by these authors? It is defined by them that "*Color-Complexity*" is to describe "*how many distinct colors are indeed contained in the test paper*". Mathematically, it is defined as occupied colors/ color unit cubes. It is a purely mathematic concept. And they discuss about the difference between human perception and mathematical color value. They have also tried to explain about the physical nature of color and concluded that RGB or HSV are the physical parameters for it. Only one literature [3] is mentioned and given for the color. It has appeared to only show that, it is color, please read. No more mention.

2) Comment 2 - The physical nature of color: In fact, the color is described exactly by the spectral reflectance in the wavelength from 380 nm to 780 nm. This spectral reflectance is dependent on the nature of material, light source, view angle, observer (the properties of eyes or camera for imaging, and the images can be reconstructed by a monitor with its technical limit) and other surrounding objects. When it is understood correctly, the color of chemical distribution is dependent on the naturally spectral reflectance of the chemical compound, the condition of light sources (day light, daylight + reflected light by other objects, the mixing of them...etc.), the technical limits of camera ($R(\lambda)$-$G(\lambda)$-$B(\lambda)$ curves of a camera), the particular R-G-B system of an applied monitor for reconstructing photos, the level of brightness, the quality of the camera sensors and so on. The total imaging process decides the quality of the photos. In that, the value of R-G-B or H-S-V are only a small indicator and the last integrated output for the imaging process and image reconstruction with the technical limits of both camera system and monitor system.

3) Comment 3: The authors say that the color identification is to identify the homogeneity of sprayed liquid reflected by the amount of chemical and spatial location.

(***a***) ***About spatial location***: The method of the author is just only 2D-location, but no 3D-location, where stereo cameras (stereo camera distance measurement) with two different imaging angles should be applied for two different axes. This point is also one of the main keys for solving the shading effect (no combination RGB and HSV data-format and scale like the mentioned conclusion, because HSV or RGB is only the conversion between color systems with the same core of the information). It is important for precision agriculture, if the authors would like to solve the real distance identification based on digital images. Hence, the 3D spatial distributions can be implemented for the correct precision agriculture so that the precision agriculture is a real precision with the 3D-homogeneity of 3D-spatial distributions.

(b) about amount of chemical that is reflected via its colors: The authors have not mentioned the imaging process of the amount of chemical under a specific light condition. Even, the applied values RGB or HSV on the test papers have not reflected entirely the nature of this process. Please authors research and explain more about it. It can be understood more in Comment 2.

4) Comment 4 and relevant questions on the color of dots and background paper: The authors say that the image dataset was collected by the researchers from GEOSTAR Technology Inc. without an evidence or a citation for them. Hence, there are many questions about the purple dots on the yellow test paper. Why must they be purple dots on the yellow test paper? What happens with other colors of the objects (called as dots) and of background (called as test paper)?

5) Comment 5 and relevant question on shading effects: what are shading effects? How is the effect of shading on the digital images? Even, not only shading, but other noises such as the changes of light sources, the surrounding-colored objects and so on? How to process these problems with the algorithms? It is obviously that shading is only the effect of a physical object (such as a tree) causing another mixing light (for example, no direct day light, but the mixing of direct daylight and reflected daylight by many colored physical objects). The mixed light can be measured and reconstructed total

exactly. Therefore, the shading can be measured and reconstructed. Therefore, its effect or affects can be determined on the digital images. For a huge amount, the machine learning can be applied but, on this principle, then total imaging process can be rebuilt quite exactly and entirely. It is the second main key to solve the shading effect and other noises (beside the mentions in (3a)).

6) Comment 6 and relevant requirements: Only 3 literatures including 1-Literature 1 for spraying, 2-Literature 2 is to show about the application of drone and 3-Literature 3 is only to say, the color is over there, if someone would like to read it, meanwhile the color is one of two main aspect of this paper. And the rest aspect is the spatial location is only 2D for "*precision*" agriculture. It is too poor and too subjective for this research approach. Even, the authors have given a strong critic on other methods when there is no similar implementation with the same test objects. The underlying comments and announcements were given very surely and strongly in hurry when the color concepts and other color methods/approaches in the combination with the appropriate updated IT-methods (machine learning, deep learning, AI, data clustering) and the physical measures of colors, light sources, light mixing, color mixing and relevant issues have been not analyzed and researched particularly.

D) Conclusion

"*Accept with major revisions*" is surely my option.
