# Supplementary material for: Color-complexity enabled exhaustive color-dots identification and spatial patterns testing in images
Source: PLoS One. 2021 May 11;16(5):e0251258. doi: 10.1371/journal.pone.0251258 (PMC8112684; doi:10.1371/journal.pone.0251258)
Supplement: Attachment — Submitted filename: Response to the Editor.pdf [file pone.0251258.s002.pdf]

1) Comment 1 - The concept "Color-Complexity": Where does this concept come from? It has just introduced at the first time by these authors? It is defined by them that "Color-Complexity" is to describe "how many distinct colors are indeed contained in the test paper". Mathematically, it is defined as occupied colors/ color unit cubes. It is a purely mathematic concept. And they discuss about the difference between human perception and mathematical color value. They have also tried to explain about the physical nature of color and concluded that RGB or HSV are the physical parameters for it. Only one literature [3] is mentioned and given for the color. It has appeared to only show that, it is color, please read. No more mention.

[We will add the following information into our revised version]

[Color complexity of an image.]

Theoretically, any data set has a Kolmogorov complexity in Information Theory. This complexity is referred to the shortest length of computer program that can regenerate the data a one whole [refs]. Though this is not a computable concept, it is highly relevant to majority of data analysis involving with finding its pattern-based information content. Unlike the concept of Kolmogorov's complexity, color complex is simply considered through human's data visualization, not computer's data generation. This is a concept not yet being well established in Color Theory.

[Ref]Ming Li; Paul M.B. Vitányi (2009). *An Introduction to Kolmogorov Complexity and Its Applications*. Springer.

A colored image is a huge dataset because of its many thousands or millions of pixels, and each pixel is coded with a 3D RGB and HSV coordinates. Color complexity of an image can be perceived and computed from two perspectives: color variety and color distribution [ref]. In this paper, both perspectives are being studied across five experimental images.

Color variety is referred to how many ``distinct" colors are present within an image, while color distribution is referred to 2D spatial distributions of distinct colors on the 2D Euclidean plane of the image. We discuss these two perspectives computationally in this study.

Even though, each of the five images seemingly have only two major colors by experimental design, the color variety within the RGB space surely would be much more than 2. There are many underlying factors that could affect large and fine scales coloring of an image. We just list three key factors here as follows. First, the mechanism of light's reflection upon an unsmooth and uneven colored paper media involves with complicate physical nature of color, as would be briefly depicted with some basic facts below. Secondly, colored dots are of many sizes, and indeed could contain heterogeneous color-pigment intensities. Thirdly, swiftly varying lighting conditions could go through the lens of cameras when the images were taken. Due to these reasons and beyond, we expect that one image would give rise to a RGB point-cloud occupying many 3D coordinates within the RGB space.

We can intuitively define an image's color variety as the relative size of its RGB point-cloud on the finest scale with respect to the size 256^3 of 3D RGB space $[0, 255]^3$. Based on this definition, each of the five images would have a rather small color complexity. It is because that the two major colors would make three RGB coordinates as three features of 3D data points being highly associated. That is, all RGB point-clouds are to have a two-hill shape occupying small volumes in the RGB space.

As for the perspective of color distribution, we only focus on the targeted purple color's distribution because the Yellow color is the background color. Since the spraying mechanism is a mechanical one. So, we expect that the purple color's distribution is far away from uniform, particularly for dots of large size with high intensity of purple. But its exact distributional shape could only be captured by rather flexible spatial structures, such as minimum spanning trees (MST).

[ref] Zhou, B., Xu, S. and Yang, X-X. Computing the color complexity of images. IEEE Xplore, 2015. (2015 12th International Conference on Fuzzy Systems and Knowledge Discovery (FSKD))

2) Comment 2 - The physical nature of color: In fact, the color is described exactly by the spectral reflectance in the wavelength from 380 nm to 780 nm. This spectral reflectance is dependent on the nature of material, light source, view angle, observer (the properties of eyes or camera for imaging, and the images can be reconstructed by a monitor with its technical limit) and other surrounding objects. When it is understood correctly, the color of chemical distribution is dependent on the naturally spectral reflectance of the chemical compound, the condition of light sources (day light, daylight + reflected light by other objects, the mixing of them...etc.), the technical limits of camera (R(λ)-G(λ)-B(λ) curves of a camera), the particular R-G-B system of an applied monitor for reconstructing photos, the level of brightness, the quality of the camera sensors and so on. The total imaging process decides the quality of the photos. In that, the value of R-G-B or H-S-V are only a small indicator and the last integrated output for the imaging process and image reconstruction with the technical limits of both camera system and monitor system.

[We will add the following information into our revised version]

Human perception recognizes color categories: red, orange, yellow, green, blue and violet(purple), when different visible lights (electromagnetic radiation) are received by our photoreceptor cells, including the three types of cone cell in our eyes. In physical world, the region of wavelength of the rainbow of visible lights is between 780nm to 380nm (in decreasing order at nanometer scale). That is, Red has the longest wavelength (around 700nm) and smallest frequency (428 Terahertz (THz)), while Purple has shortest wavelength (380nm) and largest frequency (714THz). The Yellow is in between with wavelength around 580nm and frequency around 517THz. Such linear ordering of wavelength and frequency of visible light is turned into a circular order of colors, called color wheel, through human perception. Isaac Newton has studied this nonlinearity in 17th century. Now we know that the three types of cone cells in our human eyes, which specifically respond to three visible lights: Red, Green and Blue, collectively generate all colors shown on a color wheel [1]-[3].

How the lights come from outside world into our eyes or lens of cameras is the topic of physical nature of color. Outside of eyes and camera, the color should be described exactly through the spectral reflectance within the wavelength region from 380 nm to 780 nm. This spectral reflectance, including diffuse reflection, depends on the nature of material and its surface properties, light source, viewing angles, observer (the properties of eyes or camera for imaging) and other surrounding objects. Light not only reflects from surface of material, but also penetrates beneath the surface and then scatters. Blue has highest scattering intensity than that Red and Green due to its short wavelength and high frequency.

How visible lights are composed into colors inside eyes and cameras is the topic of human trichromacy. The RGB color space is resulted from technical convolution of three monochromatic spectral stimuli: R(λ), G(λ), B(λ), curves [1]-[3].

[Refs]

1) Wyszecki, Günther; Stiles, W.S. (2000). Colour Science: Concepts and Methods, Quantitative Data and Formulae (2nd ed.). New York: Wiley Series in Pure and Applied Optics.
2) R.W.G. Hunt (2005). *The Reproduction of Colour* (6th ed.). Chichester UK: Wiley–IS&T Series in Imaging Science and Technology.
3) Schwartz MD. Lecture 17: color;. Available from: https://scholar.harvard.edu/files/schwartz/files/lecture17-color.pdf.

3) Comment 3: The authors say that the color identification is to identify the homogeneity of sprayed liquid reflected by the amount of chemical and spatial location. (a) About spatial location: The method of the author is just only 2D-location, but no 3D-location, where stereo cameras (stereo camera distance measurement) with two different imaging angles should be applied for two different axes. This point is also one of the main keys for solving the shading effect (no combination RGB and HSV data-format and scale like the mentioned conclusion, because HSV or RGB is only the conversion between color systems with the same core of the information). It is important for precision agriculture, if the authors would like to solve the real distance identification based on digital images. Hence, the 3D spatial distributions can be implemented for the correct precision agriculture so that the precision agriculture is a real precision with the 3D-homogeneity of 3D-spatial distributions. (b) about amount of chemical that is reflected via its colors: The authors have not mentioned the imaging process of the amount of chemical under a specific light condition. Even, the applied values RGB or HSV on the test papers have not reflected entirely the nature of this process. Please authors research and explain more about it. It can be understood more in Comment 2.

[We will add the following information into our revised version]

The authors strongly agree with the reviewer's comment on employing stereo cameras with more single imaging angle. Such Data can reveal truly 3D-homogeneity of 3D-spatial distributions. Analysis of such 3D distribution data can resolve many realistic and essential issues facing precision agriculture, that are unlikely could be solved by combinations of RGB and HSV data of 2D images. However, since we are indeed limited to this 2D image-format in this paper, it is data analysts' job try our best to extract as much information as we can from observed data sets. Given the RGB and HSV are two distinct aspects of same images, they provide slightly distinct pattern information. Therefore, it is natural to combine both aspects of pattern information in hope of improving on results relying on solo representation.

4) Comment 4 and relevant questions on the color of dots and background paper: The authors say that the image dataset was collected by the researchers from GEOSTAR Technology Inc. without an evidence or a citation for them. Hence, there are many questions about the purple dots on the yellow test paper. Why must they be purple dots on the yellow test paper? What happens with other colors of the objects (called as dots) and of background (called as test paper)?

[We will add the following information into our revised version]

The yellow paper is Teejet water sensitive paper (part# 20301-1N). It has been used to evaluate spray distribution, swath widths, droplet densities and penetration of spray by many UAV companies. This yellow paper is stained blue-purplish by exposure to aqueous spray droplets. More information on the design of UAV machine and nozzle's spray angle-vs-coverage relational information is contained in page-4 of the following document:
https://www.teejet.com/CMSImages/TEEJET/documents/catalogs/technical_information-m.pdf (p.4).

At this stage, any information of physical nature of color upon this paper media is seemingly not existing to our best knowledge. It is certainly worth studying it in near future due to its widely use.

5) Comment 5 and relevant question on shading effects: what are shading effects? How is the effect of shading on the digital images? Even, not only shading, but other noises such as the changes of light sources, the surrounding-colored objects and so on? How to process these problems with the algorithms? It is obviously that shading is only the effect of a physical object (such as a tree) causing another mixing light (for example, no direct day light, but the mixing of direct daylight and reflected daylight by many colored physical objects). The mixed light can be measured and reconstructed total exactly. Therefore, the shading can be measured and reconstructed. Therefore, its effect or affects can be determined on the digital images. For a huge amount, the machine learning can be applied but, on this principle, then total imaging process can be rebuilt quite exactly and entirely. It is the second main key to solve the shading effect and other noises (beside the mentions in (3a)).

[We will add the following information into our revised version]

The lighting conditions of mixing light likely, if not typically, cause uniform shading effects across the entire image under analysis. Such uniform effects can be dealt with by taking off via various imaging processing techniques. We certainly would be more than happy to take off the shading effects entirely or at least reduce such effects to the minimum as data pre-processing techniques. Nonetheless we have not performed such data pre-processing step. The simple reason is visible and explainable when look at these five images simultaneously in this paper.

In contrast, we see that the shading effects seen and considered in this paper are primarily local and heterogeneous. They are mainly caused by unevenness of the test paper, or some surrounding objects, such as human hands holding the test paper, that indeed block lights from shining onto small parts of the test paper when it was posted for its images to be taken. Such characteristics of locality and heterogeneity on an image is different from the uniform ones.

Our machine learning based approach is proposed to identify localities affected by the shading effects and to figure out the varying degrees of the effects in a divide-and-conquer fashion. Further, such local and heterogeneous characteristics of shading effects vary from one image to another image. Therefore, we need at least a learning region being anchored for each image to train our computing methodologies on typical areas, and then modify our learned methodologies one way or the other to adopt to shading effects upon affected localities. We believe that the local and heterogeneous nature of shading effects, as being present across all five images studied here, are not uncommon in real world applications.

6) Comment 6 and relevant requirements: Only 3 literatures including 1-Literature 1 for spraying, 2-Literature 2 is to show about the application of drone and 3-Literature 3 is only to say, the color is over there, if someone would like to read it, meanwhile the color is one of two main aspect of this paper. And the rest aspect is the spatial location is only 2D for "precision" agriculture. It is too poor and too subjective for this research approach. Even, the authors have given a strong critic on other methods when there is no similar implementation with the same test objects. The underlying comments and announcements were given very surely and strongly in hurry when the color concepts and other color methods/approaches in the combination with the appropriate updated IT-methods (machine learning, deep learning, AI, data clustering) and the physical measures of colors, lightsources, light mixing, color mixing and relevant issues have been not analyzed and researched particularly.

We humbly accept the reviewer's comments on shortcomings revealed through our writing in this paper. These comments are surely very well taken. As the matter of fact, these comments are very helpful toward the authors who are all relatively new in this essential research area.

In this revised version, we try our best to accommodate all reviewers' major and minor comments that are within our capabilities. This version is now clearly divided upon the two perspectives of Color Complexity. The computational developments and comparisons become rather expanded. We have added many references wherever we find necessary. We conduct new experiments to show that our proposed color identification approach is favorably compare with existing ones. We also perform simulation experiments to demonstrate our computing approaches are proper for addressing the two perspectives of color complexity.

Certainly, there are many issues raised by reviewers, such as light sources, light mixing, color mixing and relevant issues, are currently beyond our data as well as our capabilities. As data analysts, we know that we need to expand our domain knowledge to do well in any domain sciences. We humbly remember these comments and hope that these comments would guide our research in this interesting and important area in the near future.
